# Multiple Agricultural Production Efficiency in Horro District of Horro Guduru Wollega Zone, Western Ethiopia, Using Hierarchical-Based Cluster Data Envelopment Analysis

**DOI:** 10.1155/2022/4436262

**Published:** 2022-05-16

**Authors:** Tolesa Tesema, Bacha Gebissa

**Affiliations:** Lecturer at Department of Agricultural Economics, Wollega University, P.O. Box 395, Nekemte, Ethiopia

## Abstract

Multiple agricultural productions were a way of life for Ethiopian farmers. However, it was known for low productivity due to improper resource allocation. Hence, the farm household is under food insecurity and earned a low annual income. To overcome these challenges, the present study used hierarchical-based cluster data envelopment analysis by collecting data from 152 sample households through structured questionnaires. The finding suggested that the farm households in the study area were characterized by the low level of technical efficiency in multiple agricultural productions, implying that most farmers were unable to keep up with the current production frontier and technologies. The study's key result is that hierarchical-based cluster data envelopment analysis is more efficient than traditional data envelopment analysis. Furthermore, farmers in the study area are technically inefficient. From the determinants of technical efficiency in multiple agriculture, access to credit and the fertility of farmland have a positive impact on technical efficiency, whereas the age of the household and distance from infrastructures have a negative impact. Based on the significant determinants of efficiency, the present work recommends government agencies and agricultural development planners must improve farmers' knowledge towards soil fertility management practices through the construction of soil bunds, tree planting, grass planting, fencing, and the use of natural fertilizer; expansion of microfinance to rural area; and construction of the road for the market facility in the study area. Additionally, changing farmers' knowledge towards the uses of integration of manure products from livestock as fertilizer inputs for crop production and residues of crops as livestock consumption were paramount important.

## 1. Introduction

In Ethiopia, crop and livestock subsector contributes 27.4% and 7.9% to gross domestic product, respectively [1]. However, in the least-developed countries, including Ethiopia; agriculture is mostly subsistence, with different agricultural outputs supplying the majority of domestic products to farm households [2]. Agricultural progress in the past was based on land expansion, increased use of new technologies, and expansion of extension services to meet these subsistence needs [3, 4]. Nonetheless, in the current condition of a least-developed country with a low living standard, it is difficult to expand and adopt new technologies to meet national needs [5]. As such, productivity improvements through technical knowledge can advance the economies of farmers as sources of competitive advantages and intensive uses of input [6]. Thus, improving multidimensional agricultural production efficiency is a critical policy concern for agricultural advancement and evidence for making informed decisions on how to allocate limited resources [7]. Agricultural farming with multiple crops and animals increases technical efficiency more than crop or animal specialization in case crops and animals complement each other with little competition [8]. Hence, to achieve sustainability of complementarities between crops and livestock at the farm level, increased efficiency resources used, enhanced production management, and lower production costs were important [9–11]. As a result, greater extensive help for rural smallholders' livelihood is provided via rational strategic decision-making based totally on integrated crop and livestock, which increases resource use efficiency and aids development plan [12, 13]. Additionally, multiple crop-livestock systems also generate higher economic efficiency in saving production costs through complementarities between crop and livestock. To assess these concerns, the majority of empirical research in Ethiopia were estimating efficiency [14–17]. However, above-mentioned researchers analyze efficiency for single crop, and they used stochastic frontier, which is the only model used for single crop or livestock. Additionally, dynamics in social development cause the efficiency differential. Thus, study in one place does not determine other place. Hence, efficiency analysis is a paramount important gap in such dynamic environments. Moreover, current research uses data envelopment analysis that is applicable for multiple agricultural production efficiency. In data envelopment analysis efficiency assessments weights for inputs and outputs are estimated to be the best advantage for each unit to maximize relative efficiency [18]. Even if farmers in the study area are engaged in the production of crop and livestock, the productivity of both sectors is low. Farm efficiency study helps determine the level to which farmers are using the existing technologies efficiently, the potential for raising output with the existing technology, and eventually the possibility to raise productivity. Hence, the current research examines a problem setting that is somewhat related to multiple agricultural efficiencies in the form of a hierarchical structure that considers more than six different crops such as wheat, barley, teff, maize, Niger seed, bean potato, and pea and also the combination of two animal products such as poultry and milk at the same time in the study area. Additionally, on determinants of multiple agricultural production efficiency, research also introduces new variables, such as how soil fertility can be increased by the construction of a soil bund, tree planting, grass planting, fencing, and the use of natural fertilizer. In addition, by using a hierarchal-based cluster-adjusted data envelopment estimator approach, this research presents one way to improve the data envelopment model. Moreover, current research analyzed the levels of multiple agricultural production efficiency through resource allocation and identifying the determinants of to support development in more intensified form in the study area in particular and for policy makers in general. Therefore, the present study analyzes the levels of technical efficiency in multiple agricultural productions and their determinants in the study area.

## 2. Literature Reviews

### 2.1. Empirical Reviews on Efficiency Analysis

Farmers in various agroecologies show varying levels of farm efficiency while engaged in multiple agriculture productions [19, 20]. The ratio of the unit output to the maximum feasible output gives a measure of efficiency; a production frontier reveals the highest output that may be achieved under various input combinations [21]. Farmers' socioeconomic, farm, and institutional features, as well as resource ownership characteristics, have to be improved in order to increase productivity efficiency [22]. The main goals of efficiency analysis are to estimate underlying production technology and to measure household-specific technical inefficiency [23]. In the condition when the dissemination of new technologies is not yet an appropriate, collaboration is required between farmers, researchers, and technology providers to increase the efficiency of farm household [24]. One of the methods of efficiency measurements is the non-parametric method. The advantage of the non-parametric approach is that no functional form is imposed on the data, while its disadvantage lies in its assumption of constant reruns to scale and susceptibility of the frontiers to extreme observations [25]. In addition, the data envelopment frontier is both non-parametric and non-stochastic since it does not impose any a priori parametric restrictions on the underlying frontier technology (because it does not necessitate any functional form to be specified) and does not require any distributional assumption for the technical inefficiency term. Therefore, the model avoids the imposition of unwarranted structures on both the frontier technology and the inefficiency component that might create distortion in the measurement of efficiency [26]. The constant return to scale assumption is only appropriate when all firms are operating at an optimal scale. The case of different constraints may cause a firm to be not operating at optimal scale. The use of the constant return to scale specification confounded with scale efficiency when not all firms are operating at the optimal scale in measure of technical efficiency. The shortcoming of scale efficiency is that the value does not indicate whether the firm is operating in an area of increasing or decreasing returns to scale [27]. Particularly, the main criticism of data envelopment analysis is that it assumes all deviations from the frontier are due to inefficiency, and because of this, the non-parametric frontier methodology may overstate inefficiencies, and hence, outliers may have a profound effect on the magnitude of inefficiency [28]. Moreover, in data envelopment, no account is taken of the possible influence of measurement errors and other noise upon the frontier. All deviations from the frontier are assumed to be the result of technical inefficiency [29]. The reviews on efficiency analysis are summarized in Table 1.

### 2.2. Conceptual Framework

**Figure 1:**
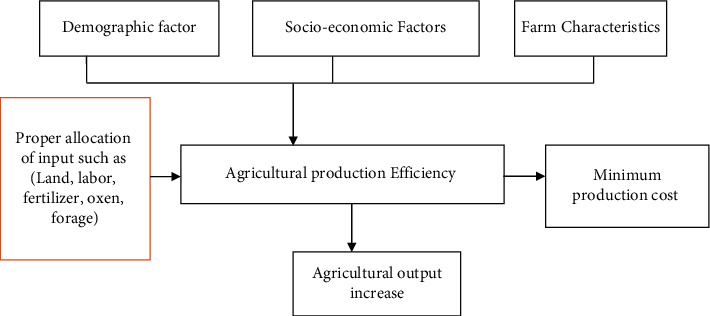
Conceptual framework of the study. Source: The authors' own design.

The input of agricultural factor endowment can alleviate the relative poverty in rural areas [40]. The efficiency with which inputs are translated into outputs is determined not only by the inputs used but also by the farmer's decision-making methods when combining these inputs. Production efficiency was determined by hosts of socioeconomic and institutional factors, as well as farm characteristics [24, 41]. The conceptual framework is used to depict how many factors interact to influence smallholder farmers' crop-livestock efficiency study area (Figure 1). As a conceptual framework Figure 1, this scenario was shown blow in a visual style to guide this research investigation.

## 3. Material and Methods

### 3.1. Description of the Study Area

The Horro district is located in the Horro Guduru Wollega zone, which is one of the zones of the Oromia national regional state. The mean annual rainfall of the district is 1,566 mm. The mean temperature is about 16.6 C, and the minimum temperature is 10.78 C. Whereas the maximum temperature was 22.32 °C. Agroecology of the district is dega (43%), woina dega (55.56%), and Kola (1.24). Horo woreda is situated at a distance of 314 km, western of Finfine, the capital city of Ethiopia; geographically, it is located between 9 9°34ʺ12 N and 37°6ʹ0ʺN. The district is bordered by the Jardaga Jarte district in the north, Jimma ganati district in the south and southeast, Abe Dongoro district in the north, and Abay-coman district in the east. The district has the total land area of 96,638.8 km^2^.

### 3.2. Sample Size and Data Used

For the present study, a total of 152 households were surveyed to collect both qualitative and quantitative data. In the first stage, the Horro district was selected from the Horro Guduru Wollega zone purposively since the researcher is convenient to the area. In the second stage, 3 kebeles, namely Didibe Kistana, Loti Ano, and Gitilo Horro, were selected from Horro district. In the third stage, 152 households from 3 kebeles were selected for the survey (Table 2). Before collecting the data, permission from the district office of agriculture and natural resources was obtained. However, owing to the country's political circumstances in general and the research area in particular, a written agreement was not permitted. However, pertinent information were obtained for the current study since researchers collect it by explaining the need for research to different stakeholders such as the woreda agricultural office, kebele leaders' office, and development agents of districts. We modified questionnaires at the office before collecting data from sample households. During data collection, primary data information was acquired at the household level using a structured questionnaire. The information gathered includes farm output, production inputs used in the production process, socioeconomic aspects, and various farm features.

The sample size for this study was determined based on the following formula given by Yemane (1967):

(1) $$\begin{array}{c} n=\frac{N}{1+\left(e^{2}\right)N}, \end{array}$$

where *n* is the sample size, *N* is the number of households (5,703), and *e* is the desired precision level. An accuracy level of 8% is used.

### 3.3. Analytical Model Specification

The ratio of total production to total annual expenditure has traditionally been used to measure agricultural efficiency [42]. Without predetermined functional forms or distributional assumptions, data envelopment analysis calculates efficiency analysis under alternative technology [43]. The benefit of a non-parametric technique is that no functional form is imposed on the data, but the disadvantage is that it assumes a constant return to scale and that the boundaries are susceptible to extreme observations [44]. Because there are two dependent variables (crop and livestock) in this study, data envelopment analysis was used. The input set, *L*, represents the technology that converts many inputs into multiple outputs (crop outputs and livestock output; *y*). *L*(*y*) is the input set such as land, labor, oxen, and material inputs that satisfies constant returns to scale and strong input disposability:

(2) $$\begin{array}{c} L\left(y\right)=\left(x:y\text{ can produce }x\;x\in R+,Y\in R+\right). \end{array}$$

In multiple agricultural productions, farmers have more control over inputs than outputs; hence, an input-oriented data envelopment analysis paradigm was chosen. The technical efficiency measure, also known as the total technical efficiency constant return to scale measure, can be calculated using the constant return to scale data envelopment framework as follows:

(3) $$\begin{array}{c} \max E_{0}=\frac{\sum_{=0}^{4}m_{r}y_{r^{0}}}{\sum_{=1}^{n}s_{i}x_{i^{0}}}\\ \text{subject to}\;\frac{\sum_{=0}^{4}m_{r}y_{r^{0}}}{\sum_{=1}^{n}s_{i}x_{i^{0}}}, \end{array}$$

where *y*_*rj*_ and *x*_*ij*_ are the *r*^th^ output such as values of crop and livestock produced and *i*^th^ input (land, labor, oxen, and material inputs), respectively, of the *j*^th^ farmers; *m*_*r*_ is the weight given to the *r*^th^ output; *s*_*i*_ is the weight given to the *i*^th^ input; and *e* is a small non-Archimedean number [45] for preventing the farmers to assign a weight of zero to unfavorable factors. This fractional program is computed separately for each farmer to determine its best possible input and output weights. In other words, the weights in the objective function are chosen to maximize the value of the farmer's efficiency ratio and meanwhile respect the less-than-unity constraint for all the farmers in the study area.

The data envelopment analysis methods have a weakness in that it implies that all decision-making units are homogeneous and operate in the same way. However, the efficiency of subunits inside a plant is currently a source of worry. If the data envelopment analysis model is used to analyze the diverse decision-making units without any adjustments, the data envelopment analysis model will produce biased performance scores and erroneous analyses. As a result, this research used a new algorithm called clustering-based technique to compute the data envelopment analysis model in non-homogeneous decision-making units. Clustering is the process of organizing a data set into a useful set of mutually exclusive clusters such that the similarity of the objects within a cluster is maximized while the assumption that the decision-making units are operating in a similar environment is minimized in classical data envelopment analysis [46, 47]. Tobit models were applied to estimate factors that affect technical efficiency differentials in multiple agricultural productions. Three marginal effects were computed from the Tobit model such as total change, expected change, and changes of probability. Estimation with ordinary least square regression of the efficiency score would lead to a biased parameter estimate since ordinary least square regression assumes normal and homoscedastic distribution of the disturbance and the dependent variable [48]. To overcome these problems, this study employed Tobit regression that is specified as follows:

(4) $$\begin{array}{c} E_{i}*=\delta o+\delta_{m}Z_{im}+v, \end{array}$$

where *E*_*i*_*∗*− the technical efficiency scores of farmers in multiple agriculture, *m*− the number of factors affecting efficiency, *δ*− a vector of the parameter to be estimated, *Z*_*im*_− farm-specific factors affecting the efficiency of *i*^th^ farm such as access to credit, livestock owning, age of household, fertility perception, sex of household, distance to market, off-farm income, extension contact, family size, and education levels, and *μ*− an error term that is independently and normally distributed with zero mean and variance *δ*^2^.

### 3.4. Measurements of Variables and Their Hypothesis

Output variables: During the production period, agricultural outputs that sample households produce were collected, and their relative prices were utilized to calculate the value of farm output. Crop output was Wheat, Barley, Teff, Maize, Niger Seed, Potato, Bean, and pea while livestock output was poultry and milk. These outputs were multiplied by their relevant market price to obtain the output value calculated from the average price of a sample household.

#### 3.4.1. Production Inputs and Costs

  Land: The amount of grazed and farmed land in hectares allotted to multiple agricultural productions during the 2019/2020 production season was expected to have a significant impact on output.
  Labor: It refers to sample households' use of family and hired labor for various crop production and livestock keeping. Conversion factors for different labor classes involved in the production process were used to convert it to man-equivalent.
  Oxen: It represents the amount of oxen used for crop production measured in oxen-day.
  Material inputs: Veterinary, feed, nitrogen phosphorous sulfur, UREA, seeds, and chemicals used by the farm household during the production period were included.

#### 3.4.2. Measurements of Determinants' Efficiency and Their Hypothesis

Uses of credit: This variable was measured as a dummy variable with a value of 1 if the household uses the credit throughout the production season and 0 if it does not use. The reason the researchers measure as dummy is the amount of credit provided for smallholder farmers in the study area is almost the same that was given by microfinance institution Oromia Credit and Saving Company. Moreover, the ratio of farmers who were not receiving the credit was much in the study area.
Livestock ownership: This variable represents the farmers' total livestock holdings in tropical livestock units during the production year.
Age of household head: This is the age of the household head in years, which were measured as a continuous variable with a negative relationship to technical efficiency.
Perception of farmers on fertility status of farmland: This variable is measured as dummy variables measured by perception of farmers, with 1 if farmers perceive their farmland as fertile and 0 if they perceive it as infertile. The farmers were the ones who were cultivating their land year by year; thus, they were able to perceive the fertility of their farmland.
Sex of the household head: This was measured as a dichotomous variable that takes a value of 1 if the household head is male and zero, if female.
Distance from market: This was the remoteness of the household head from available infrastructures in minutes.
Income from off-farm activities: This variable was measured as a dummy variable that takes a value of 1 if a farmer is received the income from off-/non-farm activities and 0 if not received income from off-farm activities during the production year.
Extension contact: This variable was measured as a continuous variable refers to the frequency of contacts with extension workers in a production period.
Family size: This variables was measured as family size of households measured in man-equivalent during the farming year.
Education level: The household head's education was measured in years of completing formal education.
  1. In order to collect data for output for each crop, the measurement approach is different. The authors collect by measurement in local area and change to the standard unit. One kuna is 10 kilograms that is equal to 0.1 quintal for a crop, and the amount in liters was collected for milk.
  2. Since the government owns the land, there is no formal land market for purchase and sale. Thus, renting, sharecropping, and borrowing of land were used as proxy variables for the price of land. One qoti is around a quarter of a hectare in the study area.

## 4. Results and Discussion

The result section contains a description of both crop and livestock products and an analysis of efficiency level using data envelopment analysis and its determinants using the Tobit model.

### 4.1. Description of Multiple Agricultural Output Produced by Sample Households

In the study area, wheat, barley, teff, maize, Niger seed, faba bean, potato, and pea were the principal crops produced during the production year, according to data collected from 152 sample households. From that crop area, wheat occupies the largest portion of the household's total cultivated land, accounting for 0.5 hectare (29.41%). Second, for Niger seed, maize, and teff, the household allocated 0.22, 0.21, and 0.19 hectares, respectively. For barley production, the sample household allocated 0.17 hectare. Finally, 0.167, 0.148, and 0.065 hectares were allocated to faba bean, pea, and potato, respectively (Table 3). Given the differences in mean productivity among crops, the survey results show that the sample household received 18.6 quintals of wheat on average, accounting for 36.37% of total production. Maize, barley, and potato output shares 12.481 (24.40%), 5.29 (10.34%), and 3.06 (12.67%) quintals of total major crop production, respectively. On average, households earned 4.89, 2.97, and 2.37 quintals of Niger seed, faba bean, and pea, respectively. During the production year, the output of livestock is collected from milk and poultry obtained by households. Finally, the average amount of milk produced was 407 liters, and the average amount of egg produced per production year was 69.92 kilograms (Table 3). As a result of the low output and limited land holding, in study area, efficiency score was low. According to the result from the data, the average amount of cropland allocated was less than 2 hectares. This shows that the farmers in the study area were small-scale farmers who produce the crop and livestock for life sustenance. This result was consistent with the findings that smallholder farmers work on plots of land that were substantially less than 2 hectares [49].

One quintal is equivalent to 100 kilograms (Crops, liters for milk production, and poultry egg produced are measured in numbers).

### 4.2. Hierarchical Clustering-Based Efficiency Scores

The data envelopment analysis yielded a mean technical efficiency score of 65.2% and 7 non-overlapping clusters were constructed based on the efficiency score (Table 4). The results of the finding show that farmers in study area are inefficient by 34.8%. This result is supported by evidence that agricultural inefficiency mainly comes from the fluctuation of pure technical efficiency, and there is low efficiency in the utilization of factor inputs.

#### 4.2.1. Group One of the Hierarchical Clusters

This cluster contains 19.73% of the total sample households. When it considers efficiency, farm households in this hierarchical cluster performed far better than other clusters. That is, through farm households' multiple agricultural production efficiency, the average technical was found to be 0.99.

#### 4.2.2. Group Two of the Hierarchical Cluster

This cluster makes up 5.92% of the overall sample household population. In this hierarchical cluster, the technical efficiency of farm households was discovered to be 0.83. When compared to the other hierarchical clusters, this cluster came in second place in terms of technical efficiency. This meant that if the typical farm household in the sample improved technical efficiency to the level of their most efficient counterpart, the average farm household could boost output by 17% using current technology.

#### 4.2.3. Group Three of the Hierarchical Cluster

Of the sample, 5.92% falls into this category. The average technical efficiency of farm households was determined to be 0.74 in this cluster. This conclusion suggested that if the average farm household in the sample achieved the same degree of technical efficiency as their most efficient counterpart, the average farm household could increase output by 26% using existing technology (Table 4). When compared to other clusters except clusters one and two, they were higher than other clusters.

#### 4.2.4. Group Four of the Hierarchical Cluster System

This cluster accounts for 21.05% of all households in the study. The average technical efficiency of farm households in this cluster was determined to be 0.67. This result showed that if the average farm household in the sample was to achieve the technical efficiency level of their most efficient counterpart, then the average farm household could experience a 33% increase in output by improving technical efficiency with the existing technology. When compared with other cluster systems, this cluster was higher than clusters five, six, and seven but lower than clusters one, two, and three.

#### 4.2.5. Group Five of the Hierarchical Cluster System

This group accounts for 20.39% of the total sample. As can be seen, the technical efficiency value coming from the average farm household was found to be 0.33. This conclusion shows that if a typical average farm household in the sample achieved the level of technical efficiency of others, the average farm household could boost output by 67% by improving technical efficiency using existing technology. When compared to other clusters, the technical efficiency of these clusters was the lowest.

#### 4.2.6. Group Sixth Hierarchical Cluster System

This cluster represents 15.13% of the whole sample. In this cluster, the average technical efficiency of farm households was determined to be 0.43. As a result, enhancing technical efficiency using existing technology might result in a 57% increase in output for a typical farm household to reach the technical efficiency level of their most efficient counterpart. When compared, this cluster was higher than cluster five and lower than other clusters.

#### 4.2.7. Group Seven of the Hierarchical Clusters

Of the sample, 18.84% is made up of this cluster. In this cluster, the average farm households' technical efficiency was found to be 0.55. When compared, this cluster was higher than clusters five and six but lower than other clusters. This implies that a typical farm household to reach the technical efficiency level of their most efficient counterpart could capability on the average 45% increase in output by improving technical efficiency, with the existing technology.

### 4.3. Determinants of Technical Efficiency in Multiple Agricultural Productions

The model LR ratio chi-square is 35.45 at degree of freedom 10 with *p*-values of 0.0001 tells as our model is significantly fit.

#### 4.3.1. Access to Credit

At a 5% level of significance, the study suggested that access to credit had a positive and statistically significant effect on technical efficiency. This is because credit enables a household to improve its efficiency, boost its ability to apply inputs, and implement farm management decisions on time. Farmers in developing countries, such as Ethiopia, pay for transportation in addition to the money paid to microfinance institutions from credit collection [50]. Furthermore, changing the dummy variable representing the household's use of credit from 0 to 1 would increase the probability of farmers being technically efficient by about 8.21% and change the average value of technical efficiency by about 5.61%, for a total increase of 2.45% in the probability and level of efficiencies (Table 5). The finding of [7] agrees with this study.

#### 4.3.2. Age of Household

At a 5% level of significance, the age of the household heads had a negative impact on the technical efficiency levels of farmers (Table 5). This indicated that younger households' technical efficiency was higher than older farmers. This could be because elderly farmers are more resistant to policy changes and information flow than younger farmers. Additionally, elder farmers fail better understanding of farming supervision including proper utilization of inputs and application of modern technologies. A unit increase in the year would reduce the probability of a farmer being technically efficient by 0.28% and the mean value of technical efficiency by about 0.19%, with an overall increase in the probability and level of technical efficiency of 0.11%, according to the computed marginal effect. The physical ability of farmers is likely to decrease as their age increases, hence decreasing their efficiency in agriculture. This finding supports the view that the ability to acquire and process useful information decrease with time [51].

Fertility status: Technical efficiency was positively influenced by land fertility status at 5% levels of significance, showing that farmers who believe their land as fertile were more likely to enhance the amount of efficiency than farmers who do not. Similarly, a change in fertility status from infertile to fertile would increase the probability of a farmer being technically efficient; the mean value of technical efficiency and overall increase by 3.08%, 6.25%, and 9.14%, according to the computed marginal effect from the Tobit model (Table 5). This research is supported by a study conducted by [52].

Distance from the market: At 1% levels of significance, distance from infrastructure has a negative impact on the sample household's technical efficiency score. This was in support of the premise that the further away farmers are from infrastructure, the more difficult it is for them to get inputs and market information. Furthermore, the estimated computed marginal effect shows that as the distance from the farm household from the market increases by 1 minute, the probability of a farmer's technical efficiency decreases by 0.43%; expected levels of technical efficiency decrease by 0.3%; and the probability and expected levels of technical efficiency decrease by 0.17% (Table 5). This finding was in line with previous research [53], which found that distance from the market is negatively connected to technical efficiency. The results of Nsiah and Fayissa [54] also found that the agricultural sector infrastructure is the main drive for agricultural production efficiency in the least-developed countries. In addition to the distance from the market, farmers have fear of COVID-19 to buy inputs from the market.

## 5. Conclusion

The main conclusion emanated from the findings of the farmers in the study area was inefficient in technical efficiency of multiple agricultural productions to achieve the maximum output frontier. As a result, the study recommends that farmers in the study area build their capacity by receiving regular training on how to make the best use of scarce resources. The findings also show that distance from infrastructure has a negative impact on smallholder farmers' technical efficiency, raising the cost of market inputs. As a result, policymakers should consider improving road development infrastructure, reducing farmer margins during input delivery, and providing access to farmers in remote areas. According to the findings of this study, the coefficient of farmer's perception of land fertility status is a determinant factor that affects technical efficiency levels, so it is critical that rural development offices advance strategies and intervene by building soil bunds, tree planting, grass planting, fencing, and encouraging the use of natural fertilizer. Furthermore, because access to credit has a favorable impact on the efficiency of smallholder farmers, governments and microfinance institutions must establish microfinance institutions in rural areas and train farmers on how to correctly apply loans for agricultural production. In the study area in particular and Ethiopia in general, more research on comparative technical efficiency analyses across the high-, low-, and midland is required.

## Figures and Tables

**Table 1:** Determinant of efficiency variable hypothesis.

Variable	Hypothesis	Model used	Authors
Credit uses	Positive	Data envelopment analysis	Hoai. [30]
Livestock ownership	Positive	Beta regression analysis	Endalew et al. [31]
Age of household head	Positive	Stochastic frontier analysis	Wang [32]
Soil fertility	Positive	Experimental approach	Anas et al. [33]
Sex of the household head	Positive	Data envelopment analysis	Oluwatayo and Adedeji. [34]
Distance to nearest markets	Negative	Meta frontier	Cheng et al. [35]
Off-/non-farm activities	Positive	Stochastic frontier approach	Mironkina et al. [36]
Extension contact	Positive	ICT-based	Aker et al. [37]
Family size	Positive	Stochastic frontier approach	Bahta et al. [38]
Education	Positive	Stochastic frontier model	Boltianska et al. [39]

**Table 2:** Total number of sample household heads.

Name of kebele	Total	Sample proportion	Sample
Didibe Kistana	560	0.23	35
Loti Ano	1450	0.59	90
Gitilo	450	0.18	27
Total	2460	1	152

Source: Own computation.

**Table 3:** Main crops produced by sample households.

Crop type	Area allocated (hectare)		Production (quintal/liter)	
	Mean	Percentage (%)	Mean	Percentage (%)
Wheat	0.50	29.94	18.6	36.37
Barley	0.17	10.18	5.29	10.34
Teff	0.19	11.38	3.06	5.98
Maize	0.21	12.57	12.48	24.40
Niger seed	0.22	13.17	2.23	4.89
Bean	0.167	10.00	1.52	2.97
Potato	0.065	3.89	6.48	12.67
Pea	0.148	8.86	1.21	2.37
Milk			407	0.86
Poultry			68.92	0.14

Source: Descriptive model result.

**Table 4:** Cluster-based technical efficiency.

Cluster groups	Cluster weight (%)	Efficiency scores	Mean
Group one	19.73684	TE_1_	0.9919
Group two	5.921053	TE_2_	0.8376923
Group three	5.921053	TE_3_	0.7461579
Group four	21.05263	TE_4_	0.6716667
Group five	20.39474	TE_5_	0.3305909
Group six	15.13158	TE_6_	0.4383889
Group seven	11.84211	TE_7_	0.5526562

Source: Own computation.

**Table 5:** Determinants of technical efficiency from Tobit model result.

Variables	Technical efficiency		Marginal effect		
	Coefficient	Standard error	Total change	Expected change	Probability change
Access to credit	0.09238236^*∗∗*^	0.0405915	0.0820973	0.056174	−0.0245507
Livestock owning	−0.00438528	0.0035935	−0.0038907	0–0.0026607	0.0015061
Age of household	−0.00321174^*∗*^	0.0018068	−0.0028495	−0.0019487	0.0011031
Fertility perception	0.1031192^*∗∗*^	0.0396118	0.0914252	0.062517	−0.0308878
Sex of household	−0.07035714	0.0529559	−0.0616694	−0.0420375	0.0341446
Distance to market	−0.00495169^*∗∗∗*^	0.0014051	−0.0043932	−0.0030043	0.0017006
Off-farm income	−0.00226981	0.0408255	−0.0020137	−0.0013771	0.0007808
Extension contact	0.00155859	0.0021409	0.0013828	0.0009456	−0.0005353
Family size	−0.01997499	0.0141811	−0.0177222	−0.0121194	0.0068604
Education levels	−0.00649976	0.0053055	−0.0057667	−0.0039436	0.0022323
Constant	1.0457095^*∗∗∗*^	0.1302488			

*Note.* ^*∗∗∗*^, ^*∗∗*^, and ^*∗*^ refer to the level of significance at 1%, 5%, and 10%, respectively. Source: Tobit model results.

## Data Availability

The data sets used and/or analyzed during the current study will be available from the corresponding author on reasonable request.
